# Applying Spatial Analysis Tools in Public Health: An Example Using SaTScan to Detect Geographic Targets for Colorectal Cancer Screening Interventions

**DOI:** 10.5888/pcd11.130264

**Published:** 2014-03-20

**Authors:** Recinda L. Sherman, Kevin A. Henry, Stacey L. Tannenbaum, Daniel J. Feaster, Erin Kobetz, David J. Lee

**Affiliations:** Author Affiliations: Kevin A. Henry, Rutgers University, School of Public Health, Cancer Institute of New Jersey; Stacey L. Tannenbaum, University of Miami Miller School of Medicine and University of Miami Sylvester Comprehensive Cancer Center; Daniel J. Feaster, Erin Kobetz, David J. Lee, University of Miami Miller School of Medicine.

## Abstract

Epidemiologists are gradually incorporating spatial analysis into health-related research as geocoded cases of disease become widely available and health-focused geospatial computer applications are developed. One health-focused application of spatial analysis is cluster detection. Using cluster detection to identify geographic areas with high-risk populations and then screening those populations for disease can improve cancer control. SaTScan is a free cluster-detection software application used by epidemiologists around the world to describe spatial clusters of infectious and chronic disease, as well as disease vectors and risk factors. The objectives of this article are to describe how spatial analysis can be used in cancer control to detect geographic areas in need of colorectal cancer screening intervention, identify issues commonly encountered by SaTScan users, detail how to select the appropriate methods for using SaTScan, and explain how method selection can affect results. As an example, we used various methods to detect areas in Florida where the population is at high risk for late-stage diagnosis of colorectal cancer. We found that much of our analysis was underpowered and that no single method detected all clusters of statistical or public health significance. However, all methods detected 1 area as high risk; this area is potentially a priority area for a screening intervention. Cluster detection can be incorporated into routine public health operations, but the challenge is to identify areas in which the burden of disease can be alleviated through public health intervention. Reliance on SaTScan’s default settings does not always produce pertinent results.

## Introduction

Public health practitioners have mapped health data for nearly 200 years. In 1840, Robert Cowan mapped the relationship between overcrowding and fever, and John Snow’s 1854 cholera map remains famous today (1). Now, GIS (geographic information systems) is used for geocoding (assigning longitude, latitude, or other geographic indicators to street addresses) and for creating maps. Recently, epidemiologists augmented descriptive mapping with the computer applications of spatial analysis, which include 1) exploratory cluster detection; 2) adjustment for the effects of place to evaluate other risk factors; 3) quantification of the effect of place or community on disease risk; and 4) site selection for geographically targeting public health research or intervention. 

Cancer rates are routinely mapped at the county level, and visualization of geographic patterns can help researchers generate etiologic hypotheses. For instance, patterns in the 1960–1970 US Cancer Mortality Atlases prompted research that connected smokeless tobacco use with oral cancers (2) and shipyard asbestos exposure with lung cancers (3). Mapping areas with high cancer rates can help prioritize cancer control programs or prompt community interventions designed to modify risk behaviors (4). Similarly, because rates of cancer by stage can be a proxy for screening uptake, mapping geographic variation by stage at diagnosis can aid in targeting areas with low rates of cancer screening (4–9). Maps are now often used for examining geographic variation along the cancer continuum at local (ie, sub-county) levels (7,10–18).

Choropleth mapping, a common method for mapping health-related data, displays ranges of rates by geographic area; for example, the Centers for Disease Control and Prevention’s (CDC’s) state-level, interactive Behavioral Risk Factor Surveillance System maps (http://apps.nccd.cdc.gov/gisbrfss/default.aspx). However, people examining these maps cannot quantitatively assess the data because which spatial patterns are highlighted depends upon which cut points are used to create categories for mapping results. In addition, data on rates in sparsely populated areas can be outliers or statistically insignificant, leading to unwarranted alarm or inappropriate disregard (19). One solution is to aggregate data, known as “regionalization” in geography, by merging proximal data to resolve both small-number instability and potential for loss of patient confidentiality. Tools are available that restrict aggregation across physical or political boundaries or that create regions of a specific population size or similar sociodemographic characteristics (20,21). Another approach is spatial smoothing, which (much like moving averages for trends) uses neighboring data to stabilize rates in sparsely populated areas (22). However, these methods can inadvertently conceal true differences in disease rates, make cumbersome the linking with geographic data on risk factors, and obscure boundaries for high-risk areas (23,24).

Spatial analysis can detect areas, regardless of size, that have significant differences in risk. One method of spatial analysis is cluster detection: this method detects high-risk areas and tests for significance while overcoming problems related to small-area rate stability. A common cluster-detection test is the spatial scan (25,26). SaTScan software (M Kulldorff and Information Management Services Inc, Cambridge, Massachusetts) uses the spatial scan and is routinely used in public health (27). The software is funded in part by the CDC and Prevention and the National Cancer Institute. SaTScan enables epidemiologists to detect clusters with relative ease. But results are affected by which methods and parameter settings are used (27), and many researchers do not account for the effect of their selections. The objective of this article is to describe how different methodological choices in SaTScan can lead to different outcomes. To illustrate our point, we used SaTScan to detect clusters of late-stage diagnosis of colorectal cancer (CRC) in Florida.

CRC is ideal for demonstrating the use of SaTScan; it is one of the most common cancers, and mortality is mitigated, in part, by screening. Not only can routine screening reduce mortality through early detection, but types of screening (eg, colonoscopy) can result in the preemptive removal of precancerous lesions, making most CRC potentially eradicable through secondary prevention. CRC screening rates are low in Florida. In 2010, 70% of white Floridians, 64% of black Floridians, and 62% of Hispanic Floridians aged 50 or older reported having had a colonoscopy or sigmoidoscopy in the previous 5 years; 22% of white Floridians, 24% of black Floridians, and 16% of Hispanic Floridians aged 50 or older reported having had a blood stool test in the previous 2 years (28).

Because overall CRC screening rates are low in Florida, all populations in the state would benefit from increased screening. It is likely communities at high risk for late-stage diagnosis of CRC would benefit the most. In Florida, 2 populations with low screening rates and high rates of CRC deaths are blacks (Hispanic and non-Hispanic) and Hispanic whites (29), so we focus particular attention to their data in our analysis. 

## Methods for Detecting Clusters of Late-Stage Diagnosis of CRC

We conducted a population-based, ecologic study on the geographic distribution of CRC diagnosed at a late stage. The study obtained approval under expedited review from the Florida Department of Health Institutional Review Board and the Florida Cancer Registry (nos. H12005 and H12010).

We analyzed cases of CRC that were diagnosed among Floridians from 1996 through 2010 and reported to the Florida Cancer Registry. Because guidelines recommend CRC screenings begin at 50, we excluded from analysis cases diagnosed before age 50. We also excluded cases for which an autopsy report did not show CRC as cause of death. To account for changes in routine screening practices after a diagnosis, we included only primary diagnoses of CRC; however, a prior diagnosis of cancer other than CRC was not grounds for exclusion. We analyzed data on adenocarcinomas only. Adenocarcinomas, approximately 90% of all cases of CRC, arise from adenomatous polyps, and some types of screening can detect these polyps, which can be removed before they progress to cancer (30). We classified cases as early stage or late stage. Cases diagnosed in situ or at localized stage were classified as early, and cases diagnosed at regional or distant stage were classified as late (according to the Surveillance Epidemiology and End Results Summary Staging system). Because an unknown stage has a poor prognosis (35% 5-year survival rate compared with a 90% for a local stage, 70% for a regional stage, and 13% for a distant stage [31]), we classified an unstaged or an unknown case as a late-stage diagnosis.

A proprietary vendor geocoded cases to 2010 census boundaries according to the street address at diagnosis of the person with CRC. Some cases were not geocodable to a street address, and the Florida Cancer Registry does not rework these cases to identify a geocodable address, so we could not use these cases in analysis. In all, we excluded approximately 5% of the cases because they were geocoded only to a zip code and 2% because they were not geocodable even to a zip code.

We used SaTScan ver 9.1.1 in this study. SaTScan uses the spatial scan, which creates a theoretically limitless number of discreet “windows” (ie, sections) in a geographic area. The windows vary in size from the smallest (containing 1 unit of analysis, such as a census block group) to the largest (containing a user-defined maximum percentage of population to be evaluated as a cluster). Each window is evaluated as a possible cluster, and the window with the highest maximum likelihood of being a cluster is assigned a *P* value, which is adjusted for multiple testing (32). We also evaluated secondary clusters. We adjusted for the most likely clusters (*P* = .05); the maximum number of iterations, or number of potential secondary clusters, was set at 15. When using the option to evaluate secondary clusters, a primary cluster is determined and analysis is rerun, without the primary cluster data, to evaluate potential secondary clusters. This procedure produces geographically distinct clusters and a more homogenous cluster risk, and it detects potential cluster rings. For instance, the surrounding suburbs of an urban center may have lower risk than the urban center (hence the appearance of a ring), thus identifying the urban center as a potential target for prioritized intervention (33). We used circular- and elliptic-shaped scan windows simultaneously. Circular windows are best for detecting small, compact clusters and elliptic windows provide the greatest power for long and narrow clusters (34). Elliptic scans are important for states with long coastlines, like Florida, or extensive borders.

Analysis was conducted by using 2 spatial scan probability models available in SaTScan: the Poisson model and the Bernoulli model. The Poisson model detects late-stage risk clusters by using age-adjusted rates, and the Bernoulli model detects late-stage risk clusters by using a ratio of late-stage diagnoses to early stage diagnoses. We used the Poisson model to detect high- and low-risk clusters for blacks (Hispanic and non-Hispanic), Hispanic whites, and non-Hispanic whites by using US Census 2010 population data and adjusting for age and sex. We used the Bernoulli model to detect clusters for blacks (Hispanic and non-Hispanic), Hispanics whites, non-Hispanic whites, and Cubans of any race. The Cuban category was not mutually exclusive from other categories; the majority of Cubans were also classified as Hispanic white. We evaluated Cubans separately because they are an important demographic group in Florida, and the Florida data shows they are at higher risk of late-stage diagnosis of CRC. Census data for the Cuban population from the Census were not available at the level of detail necessary for the Poisson model. The Bernoulli model requires only case-level (cancer registry) data, so we used the Bernoulli model for Cubans by using the variable “Hispanic origin.”

A modifiable area unit problem (MAUP) is a situation that arises when results change at different sized units of analysis (eg, block group, census tract, county), referred to here as aggregation, or maximum cluster size, referred to here as scale. MAUP can be caused by zonation effects or by regional or contextual effects. An example of a zonation effect is when no associations are found at the county level but are found at the smaller, demographically more homogenous census-tract level. An example of a regional or contextual effect is when a county analysis does not show a trend, but a national analysis shows a north–south trend by state. To address MAUP, we conducted a series of scans at different scales: 1%, 2%, and 5% to 50% (at 5% increments) of the population at risk as maximum cluster size. At 1% scale, the maximum cluster size (or window size) evaluated as a cluster is 1% of the total population for each racial/ethnic group. The largest scale possible is 50%. Evaluating a cluster larger than 50% of the population it not an option because such a cluster would indicate areas of statistically lower rates outside the circle rather than inside the circle; although both high and low rates can be evaluated. (33). We repeated these scans using 2 levels of geographic aggregation for which census population data was available. We used census tracts (subdivisions of counties ranging from about 3,000 to 7,000 people) and block groups (smallest subdivision of a tract for which the census provides population data by age and sex with an average of 1,500 people).

We evaluated sensitivity by using a known cluster in rural Union County, Florida. A correctional facility in Union County processes new inmates from 2 of 3 state regions and provides medical care to the inmates. The constant influx of inmates into the numerator (due to daily prisoner intake) but not the denominator (which is based on the decennial census and is a “snapshot” of the population at one point in time) generates high rates of cancer. In 2011, the rate of CRC in Union County was 182.7 per 100,000, far exceeding the state average of 32.7 (35).

## Comparison of Results According to Methods Used

We analyzed 36,094 cases of CRC: 3,780 were black; 3,488 were Hispanic white; 28,826 were non-Hispanic white; and 1,501 were Cuban (Table 1). Multiple, iterative scans were computer and time intensive. The block group analysis exceeded the computing capacity of a 2GB-RAM computer. To complete analysis, we used a computer with an 8GB-RAM memory and 64-bit Java (instead of the 32-bit default). The differences in *P* values resulting from 999 versus 9,999 simulations were inconsequential, so we used 999 simulations to reduce analysis time. We also compared Monte Carlo and Gumbel-based *P* values and found minimal differences (Appendix). The use of Gumbel distributions produces more precise *P* values, increases power (36), and reduces analysis time.

**Table 1:** Case Characteristics, Colorectal Cancers Diagnosed 2006–2010 Among Florida Residents

Characteristic	Cuban^a^		Hispanic White^b^		Non-Hispanic White		Hispanic and Non-Hispanic Black^c^	
	Total Cases in Registry	Cases Selected for Study	Total Cases in Registry	Cases Selected for Study	Total Cases in Registry	Cases Selected for Study	Total Cases in Registry	Cases Selected for Study
**Total no. of cases**	2,036	1,501	4,938	3,488	39,028	28,826	5,688	3,780
**Men, % of cases**	53.3	54.8	51.9	51.6	52.1	51.9	49.8	49.4
**Stage at diagnosis, % of cases**								
Late stage	56.8	54.3	51.9	51.3	49.5	52.1	52.7	59.0
Unknown or unstaged	6.8	3.5	10.1	5.0	9.0	4.8	9.1	4.9
**Mean age of cases, y**	69.2	71.6	67.2	70.6	70.3	72.4	63.7	67.4
**Age, % of cases**								
≥50	91.9	NA	88.0	NA	92.7	NA	85.4	NA
≥65	67.8	74.6	61.8	70.3	68.4	74.0	48.3	57.4
≥75	39.4	43.5	34.5	38.8	42.4	45.8	23.5	27.5
**Diagnosis of adenocarcinoma, %**	92.2	NA	90.0	NA	90.0	NA	88.9	NA
**Autopsy did not indicate colorectal cancer, %**	<.001	NA	<.001	NA	<.001	NA	<.001	NA
**Year of diagnosis, % of cases**								
2006	23.1	24.5	19.7	19.6	21.5	21.4	19.8	19.3
2007	21.6	21.7	19.4	19.4	21.1	21.0	19.3	19.0
2008	21.4	20.5	20.6	19.8	20.6	20.6	20.5	21.3
2009	17.9	18.2	20.4	20.7	19.0	19.0	21.1	20.0
2010	16.0	15.2	19.7	20.4	17.8	18.0	19.3	20.3

Abbreviations: NA, not applicable because of case selection criteria.

a This racial/ethnic category is not mutually exclusive from the other racial/ethnic categories in this table. Most Cubans in this study were white, white Cubans were counted also as Hispanic whites, and black Cubans were counted as Hispanic blacks.

b Includes white Cubans.

c Includes black Cubans.

Table 2 summarizes cluster results by race/ethnicity, method, scale, and aggregation. For areas with identified clusters, Table 2 identifies a generic location label and reports the relative risk and *P* value for each cluster. Table 2 also reports a range and standard deviation for the magnitude of relative risk for the individual census tracts contained in the clusters — an indication of how homogenous the risk is throughout the cluster.

**Table 2:** Example Summaries of Clusters of Late-Stage Diagnosis of Colorectal Cancer, by Method, Scale, and Aggregation, Florida 2006–2010^a^

Scale, (%)	Location		Cluster		Local		*P* Value				
			Block Group	Census Tract	Block Group, Range (SD)	Census Tract, Range (SD)	Block Group		Census Tract		
**Black Cluster Summary (Bernoulli Method)**											
1	Area A		No cluster	1.60	No cluster	0–1.7 (0.8)	No cluster		.18		
2			1.55	1.48	0–1.7 (0.6)	0–1.7 (0.8)	.10		.06		
5			1.37	1.43	0–1.7 (0.7)	0–1.7 (0.8)	.04		.05		
10			1.37	1.43	0–1.7 (0.7)	0–1.7 (0.8)	.04		.05		
15			1.37	1.43	0–1.7 (0.7)	0–1.7 (0.8)	.04		.05		
20			1.37	No cluster	0–1.7 (0.7)	No cluster	.04		.05		
25			1.37		0–1.7 (0.7)		.04		No cluster		
30			1.37		0–1.7 (0.7)		.04				
35			1.37		0–1.7 (0.7)		.04				
40			1.37		0–1.7 (0.7)		.04				
45			1.37		0–1.7 (0.7)		.04				
50			1.38		0–1.7 (0.7)		.04				
20	Area A, plus a significantly larger region		No cluster	1.19	No cluster	0–1.7 (0.7)	No cluster		.05		
25				1.19		0–1.7 (0.7)			.05		
30				1.19		0–1.7 (0.7)			.05		
35				1.19		0–1.7 (0.7)			.05		
40				1.19		0–1.7 (0.7)			.05		
45				1.19		0–1.7 (0.7)			.05		
50				1.19		0–1.7 (0.7)			.05		
50	Area B		No cluster	1.59	No cluster	0–1.7 (0.8)	No cluster		.56		
**Black Cluster Summary (Poisson Method)**											
1	Area A subsection 1		No cluster	4.00	No cluster	1.7–6.4 (1.7)	No cluster		.03		
2			No cluster	4.00	No cluster	1.7–6.4 (1.7)	No cluster		.03		
5	Area A		1.55	1.43	0–45.3 (4.1)	0–1.7 (0.8)	.27		.05		
10			1.55	1.53	0–45.3 (4.6)	0–33.3 (4.2)	.12		.03		
15			1.51	1.53	0–45.3 (4.6)	0–33.3 (4.2)	.12		.03		
20			1.55	1.53	0–45.3 (4.6)	0–33.3 (4.2)	.12		.03		
25			1.55	1.53	0–45.3 (4.6)	0–33.3 (4.2)	.12		.03		
25			1.55	1.53	0–45.3 (4.6)	0–33.3 (4.2)	.12		.03		
30			1.55	1.53	0–45.3 (4.6)	0–33.3 (4.2)	.12		.03		
35			1.55	1.53	0–45.3 (4.6)	0–33.3 (4.2)	.12		.03		
40			1.51	1.53	0–45.3 (4.6)	0–33.3 (4.2)	.12		.03		
45			1.51	1.53	0–45.3 (4.6)	0–33.3 (4.2)	.12		.03		
50			No cluster	1.53	No cluster	0–33.3 (4.2)	No cluster		.03		
1	Area G subsection 1		No cluster	0	No cluster	0	No cluster		.26		
1	Area G subsection 2		0	No cluster	0	No cluster	.48		No cluster		
2			0	No cluster	0	No cluster	.48		No cluster		
2	Area H		0.35	No cluster	0–32.4 (2.0)	No cluster	.32		No cluster		
5	Area G		0.38	No cluster	0–32.4 (2.1)	No cluster	<.001		No cluster		
10			0.38	0.42	0–32.4 (2.1)	0–11.2 (1.2)	<.001		<.001		
15			0.38	0.42	0–32.4 (2.1)	0–11.2 (1.2)	<.001		<.001		
20			0.38	0.42	0–32.4 (2.1)	0–11.2 (1.2)	<.001		<.001		
25			0.38	0.42	0–32.4 (2.1)	0–11.2 (1.2)	<.001		<.001		
35			0.38	0.42	0–32.4 (2.1)	0–11.2 (1.2)	<.001		<.001		
40			0.38	0.42	0–32.4 (2.1)	0–11.2 (1.2)	<.001		<.001		
45			0.38	0.42	0–32.4 (2.1)	0–11.2 (1.2)	<.001		<.001		
50			0.38	0.42	0–32.4 (2.1)	0–11.2 (1.2)	<.001		<.001		
**Cuban Cluster Summary (Bernoulli Method)**											
1	Area A subsection 1		1.61	1.61	0–1.6 (0.72)	0–1.6 (0.76)	.40		.95		
2	Area A subsection 2		1.61	1.62	0–1.6 (0.56)	0–1.6 (0.76)	.61		.48		
5			1.61	1.62	0–1.6 (0.56)	0–1.6 (0.76)	.68		.53		
10			1.61	1.62	0–1.6 (0.56)	0–1.6 (0.76)	.70		.56		
15			1.61	1.62	0–1.6 (0.56)	0–1.6 (0.76)	.70		.56		
20			1.61	1.62	0–1.6 (0.56)	0–1.6 (0.76)	.70		.57		
25			1.61	1.62	0–1.6 (0.56)	0–1.6 (0.76)	.71		.57		
30			1.61	1.62	0–1.6 (0.56)	0–1.6 (0.76)	.71		.57		
35			1.61	1.62	0–1.6 (0.56)	0–1.6 (0.76)	.71		.57		
40			1.61	1.62	0–1.6 (0.56)	0–1.6 (0.76)	.71		.58		
45			1.61	1.62	0–1.6 (0.56)	0–1.6 (0.76)	.70		.58		
50			1.61	1.62	0–1.6 (0.56)	0–1.6 (0.76)	.90		.58		
**Hispanic White Cluster Summary (Bernoulli Method)**											
1	Area A		1.55	1.53	0–1.7 (0.80)	0–1.7 (0.71)	.24		.12		
2			1.55	1.53	0–1.7 (0.80)	0–1.7 (0.71)	.27		.14		
5			1.55	1.53	0–1.7 (0.80)	0–1.7 (0.71)	.29		.16		
10			1.55	1.53	0–1.7 (0.80)	0–1.7 (0.71)	.30		.16		
15			1.55	1.53	0–1.7 (0.80)	0–1.7 (0.71)	.30		.16		
20			1.55	1.53	0–1.7 (0.80)	0–1.7 (0.71)	.30		.16		
25			1.55	1.53	0–1.7 (0.80)	0–1.7 (0.71)	.30		.16		
30			1.55	1.53	0–1.7 (0.80)	0–1.7 (0.71)	.30		.16		
35			1.55	1.53	0–1.7 (0.80)	0–1.7 (0.71)	.30		.16		
40			1.55	1.53	0–1.7 (0.80)	0–1.7 (0.71)	.30		.16		
45			1.55	1.53	0–1.7 (0.80)	0–1.7 (0.71)	.30		.16		
50			1.55	1.53	0–1.7 (0.80)	0–1.7 (0.71)	.30		.16		
**Hispanic White Summary (Poisson Method)**											
10	Area A	No cluster		1.41	No cluster	0–10.0 (1.70)	No cluster		<.001	
25		1.41		1.38	0–50.7 (3.67)	0–10.04 (1.46)	<.001		<.001	
30		1.41		1.36	0–50.7 (3.67)	0–42.1 (2.72)	<.001		<.001	
35		1.40		No cluster	0–10.0 (1.29)	No cluster	<.001		No cluster	
40		1.41		No cluster	0–42.1 (2.71)	No cluster	<.001		No cluster	
45		1.40		No cluster	0–82.7 (3.85)	No cluster	<.001		No cluster	
50		1.40		1.37	0–82.7 (3.85)	0–104.4 (4.72)	<.001		<.001	
25	Area a (high risk)^b^	1.54		No cluster	0–5.0 (1.02)	No cluster	.07		No cluster	
30		1.54		No cluster	0–5.0 (1.02)	No cluster	.07		No cluster	
2	Area A subsection 1	No cluster		2.00	No cluster	0–10.0 (2.48)	No cluster		.05	
5		1.57		1.51	0–27.2 (3.41)	0–10.0 (1.90)	.02		<.001	
10		1.46		No cluster	0–27.2 (3.18)	No cluster	<.001		No cluster	
15		1.37		1.43	0–50.7 (3.57)	0–10.0 (1.52)	<.001		<.001	
20		1.43		1.36	0–50.7 (4.29)	0–10.0 (1.50)	<.001		<.001	
35		No cluster		1.31	No cluster	0–121.4 (7.77)	No cluster		.15	
40		No cluster		1.49	No cluster	0–25.7 (2.89)	No cluster		.33	
45		No cluster		1.49	No cluster	0–25.7 (2.89)	No cluster		.33	
5	Area A subsection 2	1.58		1.49	0–52.2 (6.34)	0–6.6 (1.27)	.02		.26	
10		1.58		No cluster	0–41.1 (5.10)	No cluster	.48		No cluster	
15		1.39		1.41	0–44.64 (3.43)	0–6.79 (1.09)	<.001		<.001	
20		1.41		1.40	0–16.2 (1.91)	0–4.92 (0.79)	.04		.06	
35		No cluster		1.36	No cluster	0–10.0 (1.29)	No cluster		<.001	
40		No cluster		1.37	No cluster	0–10.0 (1.25)	No cluster		<.001	
45		No cluster		1.37	No cluster	0–10.0 (1.25)	No cluster		<.001	
5	Area A subsection 3	1.71		No cluster	0–44.4 (5.52)	No cluster	.03		No cluster	
15	Area C	1.56		1.68	0–21.7 (2.96)	0–15.3 (1.05)	.12		.08	
20		1.68		No cluster	0–22.11 (3.02)	No cluster	.04		No cluster	
25		No cluster		1.53	No cluster	0–15.0 (2.01)	No cluster		.17	
30		No cluster		1.55	No cluster	0–22.0 (3.01)	No cluster		.09	
35		1.72		1.58	0–15.2 (2.04)	0–15.4 (2.07)	.05		.04	
40		1.61		1.61	0–47.3 (3.97)	0–15.7 (2.10)	.04		.01	
45		1.67		1.61	0–48.7 (4.08)	0–15.7 (2.10)	<.001		.01	
50		1.67		1.69	0–48.7 (4.08)	0–16.2 (2.17)	<.001		<.001	
20	Area a (low risk)^b^	0		No cluster	0–0 (0)	No cluster	.62		No cluster	
50		No cluster		0.17	No cluster	0–1.5 (0.35)	No cluster		.87	
1	Area I	0.23		0.24	0–142.5 (6.31)	0–227.5 (12.96)	.28	.32	
2		0.32		0.25	0–142.5 (5.73)	0–230.4 (13.10)	.06	.41	
5		0.34		No cluster	0–153.6 (6.18)	No cluster	.32	No cluster	
10		0.61		0.65	0–148.7 (5.38)	0–234.5 (7.47)	<.001	.06	
40		0.68		No cluster	0–169.70 (6.31)	No cluster	.13	No cluster	
45		0.71		No cluster	0–176.7 (6.57)	No cluster	.74	No cluster	
50		0.71		No cluster	0–176.6 (6.57)	No cluster	.74	No cluster	
10	Area J	0.66		No cluster	0–34.4 (2.41)	No cluster	.05	No cluster	

a Non-Hispanic whites are excluded from table for simplicity.

b Lower case “a” indicates a smaller risk cluster adjacent to a larger cluster.

**Figure 1:**
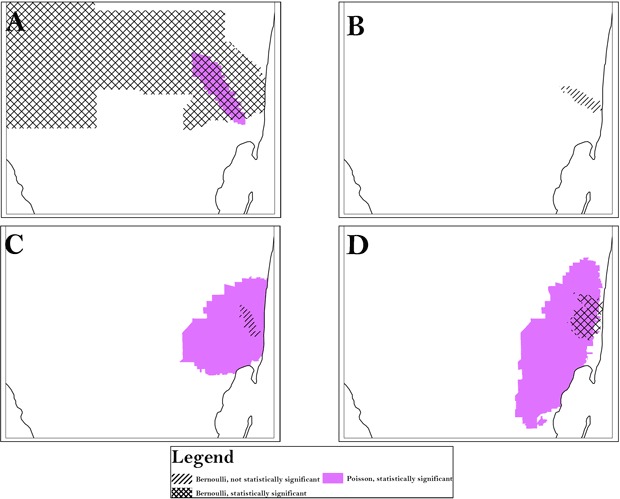
Using census tract analysis as an example, the area of persistent clusters (Area A) is indicated for all race/ethnicities and was identified by both the Bernoulli and Poisson models. A, analysis of black population; B, analysis of Cuban population; C, analysis of Hispanic white population; D, analysis of non-Hispanic white population. To preserve confidentiality, maps are presented without points of reference.

For each racial/ethnic category, we found similar clusters across scales, aggregation, and methods (Table 2). All analyses identified an area in South Florida, Area A, as high risk for late-stage diagnosis of CRC. However, the magnitude of risk was generally slightly higher at the smaller scales and at the lower levels of aggregation and often comprised greater homogeneity in local rates. For instance, for Hispanic whites, using the Poisson model, the range of relative risk for the individual census tracts that the cluster comprises is 0 to 10 at a scale of 10% with a combined risk for the cluster of 1.41. At the scale of 25%, however, the range of relative risk for the comprising tracts is 0 to 10.04 with a combined cluster risk of 1.38 and, at the scale of 30%, the range of relative risk is 0 to 42.1 with a combined cluster risk of 1.36. *P* values varied by scale, aggregation, and method, indicating clusters may be missed when a single approach is used. Using only the 50% SaTScan default or the 20% scale is often suggested, but for Hispanic whites, high-risk Area A is split into 2 smaller clusters at 20%, and low-risk Area I was significant only at the 10% scale. All scales, aggregation, and models detected high-risk clustering in Area A for all race/ethnicities, but the cluster for non-Hispanic whites extended far beyond Area A, as did the cluster for blacks found by the Bernoulli method. (Table 3, Figure 1). The Bernoulli results for Hispanic whites and Cubans were not significant, although they persisted at multiple scales and aggregations.

**Table 3:** Characteristics of Persistent Cluster Area A, Cluster-Detection Analysis of Late-Stage Diagnosis of Colorectal Cancer, by Method, Scale, and Aggregation, Florida, 2006–2010^a^

Characteristic	Black	Cuban	White Hispanic
**Significant at *P* <.10**			
No. of scales	11 of 12	0 of 12	11 of 12
Unit of aggregation (block group or census tract)	Both	Neither	Both
Method used (Bernoulli or Poisson)	Both	Bernoulli	Both
**Area selected based on**			
Scale, % of population	40	NA	50
Aggregation (unit of analysis)	Census tract	NA	Census tract
Method used	Poisson	NA	Poisson
Relative risk	1.53	NA	1.36
*P* value	.03	NA	<.001
County	Miami-Dade	NA	Miami-Dade and Broward
No. of late-stage cases	197	NA	1,652
**Demographics**			
Population total in 2010	17,036	NA	72,967
Hispanic, %	14	NA	17
Non-white, %	50	NA	54%
Below poverty, %	40	NA	31%

a Selection of area of geographic interest was based on *P* value, magnitude of risk, overlap, and evaluation of other persistent, significant clusters at that scale. Tract-level aggregation was selected to match with available area-based, sociodemographic information.

**Figure 2:**
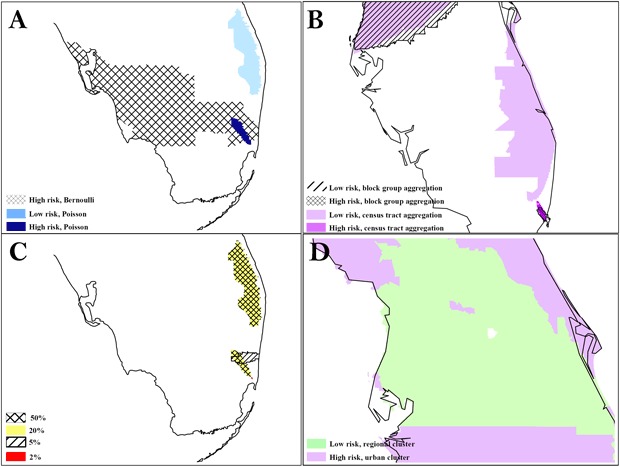
The difference in results between the Poisson and Bernoulli methods, aggregation at the census tract and block group level, and scale at 50% and 1%. A, comparison of results from Poisson vs Bernoulli methods; B, comparison of results from different units of analysis (census tracts vs block group); C, comparison of results at different scales: D, secondary cluster evaluation with an island of high risk in a region of low risk. To preserve confidentiality, maps are presented without points of reference.

At the same 50% scale for blacks, the Bernoulli method detected a cluster in South Florida that was much larger than the cluster in Area A detected by the Poisson method (Figure 2). The Bernoulli method also detected a secondary cluster in Central Florida. At the same 40% scale for Hispanic whites, both levels of aggregation detected the same high-risk cluster in the Tampa area, but only the block group analysis detected the low-risk regional cluster surrounding it. We found significant overlap between the 2 levels of aggregation in a southeast cluster, but the census tract analysis detected an adjacent, small, low-risk cluster, and the high-risk cluster detected by census tract analysis was larger. Although we detected clusters consistently at multiple scales, we also found variation, particularly for small clusters. The Bernoulli method detected clusters for blacks that had an exact overlay at the 20% and 50% scales, but the 5% scale detected only 1 partial overlay, and the 2% scale detected a small, disconnected cluster in the general area. All of these clusters were significant. The largest relative risk was at the 2% scale, and the most local risk homogeneity was at the 5% scale. Figure 2 also shows the benefit of evaluating secondary clusters; we found a significant island of high risk surrounded by a large area of low risk for Hispanic whites at the 40% scale, block group level of analysis.

The Union County cluster was identified only at the block group level for non-Hispanic whites. Block group analysis at the scales of 10%, 15%, and 20% identified the location of the correctional facility in a significant cluster (relative risk, 2.0–4.7).

## Discussion

All analyses detected an area in South Florida, Area A, as a high risk area for late-stage diagnosis of CRC and, therefore, an area that should be a high priority area for CRC screening interventions. Because both the Poisson method and Bernoulli method (which does not require population data) detected a cluster in the same general area, this cluster is unlikely to be a spurious result of denominator problems. 

Some clusters were detected consistently but were not statistically significant by any method or at any aggregation or scale. A sample size of 10,000 cases would be needed in elliptic scans to detect a significant result of a relative risk of 1.2 (25), leading us to believe that much of our analysis was underpowered. Non-significant cluster areas consistently detected at multiple scales and by multiple methods, such as the areas detected by analyzing data on the Cuban population, are commonly disregarded on statistical grounds but may warrant epidemiologic attention, particularly if the demographic composition of the population matches known risk profiles.

Incorporating cluster detection into disease surveillance can detect areas of high risk 1) to target for intervention and 2) to drive etiologic research. For screenable cancers, the Bernoulli method should be ideal for detecting communities for intervention because it detects areas at risk for late-stage diagnosis regardless of underlying rates of disease. However, using only case-level data reduces power and may miss important clusters. Evaluating the results of the Poisson and Bernoulli methods together may help detect areas with low screening rates. Communities with clusters of low risk for late-stage incidence but high risk for the ratio of late- to early-stage diagnoses may identify areas of public health importance; the low rates may be artificial, due to limited population-based screening, and these areas could be missed by using the Poisson analysis alone. However, we found this scenario only in our analysis of non-Hispanic whites.

Only the analysis of data on non-Hispanic whites detected the known cluster in Union County. That other analyses did not detect this cluster may also be a function of low power. Using higher *P* values would increase sensitivity, but ascertaining whether a non-significant cluster has public health importance may not be feasible.

Our study had numerous limitations in addition to low power. We were unable to address lag time from screening to diagnosis or any misclassification errors that may have resulted from inaccurate geocoding. We also used real-world data (not simulated data with known clusters) so we could not compare our results with a known right answer, except for the Union County cluster. Improving the quality of geocoding could reduce the rate of misclassification and increase the number of cases in the analysis, thereby reducing the potential for bias and amplify power. Correctional facilities often report cases by using post office boxes as addresses, but we excluded cases not geocoded to a street address. A review of case-level address data and eliminating duplicate records could correct this problem (an institution’s street address can be identified through Internet search). This method could improve geocoding from other reporting institutions with high rates of post office boxes, such as nursing homes, as well as inform researchers which clusters might be institutionally based clusters, that is, driven by the location of group-living facilities.

Another potential limitation is the classification of Cubans in the Florida Cancer Registry data. Cancer registries supplement the field “Hispanic origin” with information on place of birth, which is found on death certificates. Florida data on Hispanic origin has been documented to be 97% accurate (37). In our data, the percentage of people who died from CRC was highest among Cubans (33%); this rate was higher than the rate for Hispanic and non-Hispanic blacks (32%), Hispanic whites (26%), and non-Hispanic whites (32%); 18% of Cubans died from early-stage CRC, compared with 44% from late-stage CRC. Therefore, cases of early-stage CRC may have been misclassified as unknown or as general Hispanic ethnicity more often than late-stage cases. 

And, most challenging, using an iterative, multimethod approach delivered varying results. The default software setting of a 50% scale often 1) results in large clusters that are not useful for prioritizing public health resources and 2) masks small clusters that may have public health significance. But using a multimethod approach leaves the researcher without an answer to this question: where should we target screening interventions? One tactic is to target areas consistently detected through visual inspection of the maps of clusters and to use GIS overlay functions (eg, intersects) to identify areas that are consistently identified as a high-risk cluster across multiple scales, aggregation, and methods. Another tactic is to use the Gini coefficient (a measure of statistical dispersion) available in SaTScan. The larger the Gini coefficient, the greater the heterogeneity of the population; it can be applied in the same way that the coefficient of determination (*R* ^2^) is applied to aid model selection (38). Unfortunately, how to employ the Gini coefficient is not described in the SaTScan user guide. Another tactic is to use Visual Inquiry Toolkit, free software that assists SaTScan users in choosing quantitatively appropriate areas through geovisual analytics (www.geovista.psu.edu/VIT/). Unfortunately, the lack of user support and routine maintenance renders this software inappropriate for wide-scale use. 

Our study had numerous strengths. We demonstrated several methods for finding suitable locations for intensive screening for CRC. We tested those methods on a large, diverse, real-world data set, and evaluated one of the most commonly used cluster-detection software products: SaTScan. This software’s spatial scan is one of the best for power, and although it has low levels of sensitivity, it is comparable with other similar products and results in fewer false positives (25,26). SaTScan is free, was developed partially with funding from the CDC and Prevention and the National Cancer Institute, has a detailed manual and strong user support, and is maintained financially — making it an appropriate and conservative public health application for identifying target communities for enhanced screening for CRC.

No single scale or method in our study detected all significant clusters of late-stage diagnosis of CRC, and significance depended on the population size, the level of risk, and the population density of the demographic group examined. However, a perfunctory PubMed review (conducted June 30, 2013; keyword “SaTScan”; English language only; spatiotemporal scans excluded) of the 20 most recent studies that used SaTScan showed that only 3 studies used a range of scales; more than half omitted details on the scale used and any other methods used; and only 3 provided a rationale for the scale selected. This review suggests that many health researchers are unaware of the influence on results of the choice of method used for spatial analysis. Combining multiple models at different scales is appropriate for detecting areas of public health importance, but there remains a need to establish best practices for a systematic approach. Such an approach would help to ensure that clusters are “real” (ie, that the clusters are amenable to public health intervention or will contribute to etiologic knowledge). A protocol should be established so that analysis is replicable and the potential for false positives is reduced.

## Appendix. Sample of Comparisons of *P* Values Generated by Using 9,999 versus 999 Iterations and *P* Values Generated by Using Monte Carlo versus Gumbel-based Iterations^a^

**Table:**

Cluster No.	Race and Ethnicity	Model	Scale	Unit	Itera-tions	Time	*P* Value	
						(8GB-RAM, 64-Bit Java)	Standard Monte Carlo	Gumbel Based
1	HW	BM	50%	BG	9,999	8 h, 57 s	.2955000000000	.3017234034163
1	HW	BM	50%	BG	999	49 m, 3 s	.2900000000000	.3015861971047
1	HW	BM	50%	BG	999	47 0, 7 s	Gumbel only	.3015861971047
1	HW	PM	20%	BG	9,999	14 h, 46 m, 20 s	.0001000000000	.0000000321690
2	HW	PM	20%	BG	9,999	14 h, 46 m, 20 s	.0332000000000	.0339398662811
3	HW	PM	20%	BG	9,999	14 h, 46 m, 20 s	.0450000000000	.0454292744735
4	HW	PM	20%	BG	9,999	14 h, 46 m, 20 s	.6302000000000	.6253564438889
1	HW	PM	20%	BG	999	2 h, 9 m, 46 s	.0010000000001	.0000000447739
2	HW	PM	20%	BG	999	2 h, 9 m, 46 s	.0350000000001	.0386860985062
3	HW	PM	20%	BG	999	2 h, 9 m, 46 s	.0460000000001	.0440007774959
4	HW	PM	20%	BG	999	2 h, 9 m, 46 s	.6350000000001	.6173587281736
1	HW	PM	20%	BG	999	2 h, 6 m, 8 s	Gumbel only	.0000000447739
2	HW	PM	20%	BG	999	2 h, 6 m, 8 s	Gumbel only	.0386860985062
3	HW	PM	20%	BG	999	2 h, 6 m, 8 s	Gumbel only	.0440007774959
4	HW	PM	20%	BG	999	2 h, 6 m, 8 s	Gumbel only	.6173587281736
1	Black	BM	30%	CT	9,999	59 m, 56 s	.0206000000000	.0239400635312
2	Black	BM	30%	CT	9,999	59 m, 56 s	.7421000000000	.7373982545068
1	Black	BM	30%	CT	999	8 m, 43 s	.0230000000000	.0233393051534
2	Black	BM	30%	CT	999	8 m, 43 s	.7420000000000	.7335625961962
1	Black	BM	30%	CT	999	8 m, 37 s	Gumbel only	.0233393051534
2	Black	BM	30%	CT	999	8 m, 37 s	Gumbel only	.7335625961962
1	Black	PM	10%	CT	9,999	39 m, 50 s	.0060000000000	.0057729635603
2	Black	PM	10%	CT	9,999	39 m, 50 s	.0340000000000	.0326087958460
3	Black	PM	10%	CT	9,999	39 m, 50 s	.1540000000000	.1454212632260
1	Black	PM	10%	CT	999	7 m, 47 s	.0060000000001	.0057729635604
2	Black	PM	10%	CT	999	7 m, 47 s	.0340000000001	.0326087958465
3	Black	PM	10%	CT	999	7 m, 47 s	.1540000000001	.1454212632261
1	Black	PM	10%	CT	999	7 m, 45 s	Gumbel only	.0057729635604
2	Black	PM	10%	CT	999	7 m, 45 s	Gumbel only	.0326087958465
3	Black	PM	10%	CT	999	7 m, 45 s	Gumbel only	.1454212632261

Abbreviations: HW, Hispanic white; BM, Bernoulli model; PM, Poisson model, BG, block group; CT, census tract.

^a^ “Gumbel only” means only Gumble-based were run; otherwise both the default and Gumbel were run simultaneously. Times varied with network traffic as well as concurrent stand-alone computer use.
